# Similar levels of gene content variation observed for *Pseudomonas syringae* populations extracted from single and multiple host species

**DOI:** 10.1371/journal.pone.0184195

**Published:** 2017-09-07

**Authors:** Talia L. Karasov, Luke Barrett, Ruth Hershberg, Joy Bergelson

**Affiliations:** 1 Committee On Genetics Genomics & Systems Biology, University of Chicago, Chicago, Illinois, United States of America; 2 Department of Ecology and Evolution, University of Chicago, Chicago, Illinois, United States of America; 3 CSIRO Agriculture, Canberra, ACT 2601, Australia; 4 Department of Genetics, the Ruth and Bruce Rappaport Faculty of Medicine, Technion-Israel Institute of Technology, Haifa, Israel; Academia Sinica, TAIWAN

## Abstract

Bacterial strains of the same species collected from different hosts frequently exhibit differences in gene content. In the ubiquitous plant pathogen *Pseudomonas syringae*, more than 30% of genes encoded by each strain are not conserved among strains colonizing other host species. Although they are often implicated in host specificity, the role of this large fraction of the genome in host-specific adaptation is largely unexplored. Here, we sought to relate variation in gene content between strains infecting different species to variation that persists between strains on the same host. We fully sequenced a collection of *P*. *syringae* strains collected from wild *Arabidopsis thaliana* populations in the Midwestern United States. We then compared patterns of variation observed in gene content within these *A*. *thaliana*-isolated strains to previously published *P*. *syringae* sequence from strains collected on a diversity of crop species. We find that strains collected from the same host, *A*. *thaliana*, differ in gene content by 21%, 2/3 the level of gene content variation observed across strains collected from different hosts. Furthermore, the frequency with which specific genes are present among strains collected within the same host and among strains collected from different hosts is highly correlated. This implies that most gene content variation is maintained irrespective of host association. At the same time, we identify specific genes whose presence is important for *P*. *syringae*’s ability to flourish within *A*. *thaliana*. Specifically, the *A*. *thaliana* strains uniquely share a genomic island encoding toxins active against plants and surrounding microbes, suggesting a role for microbe-microbe interactions in dictating the abundance within this host. Overall, our results demonstrate that while variation in the presence of specific genes can affect the success of a pathogen within its host, the majority of gene content variation is not strongly associated with patterns of host use.

## Introduction

Many microbial species colonize diverse biotic and abiotic ecological niches [1,2]. The traits that allow a microbe to survive in these varied environments are of wide ecological, clinical and agricultural relevance. For example, many pathogenic bacteria exhibit increased virulence in their host of isolation [3,4]. There is thus widespread interest in understanding the evolutionary and genetic mechanisms that allow strains to flourish in specific environments while perishing in others.

The plethora of bacterial genome sequences that are publicly available provides broad insight into the genes that are potentially adaptive for specific hosts and environments. Numerous studies have revealed that strains of the same species collected from disparate environments differ extensively in gene content [5–9], varying in the presence of dozens to even thousands of genes. At least a fraction of this variability underlies environment-specific adaptations, and the challenge has now become determining which genes are adaptive for which environments.

It is particularly important to understand the adaptive significance of gene content diversity in the ubiquitous plant pathogen *P*. *syringae*. *P*. *syringae* is a genetically diverse bacterial species complex encompassing lineages with both pathogenic and non-pathogenic lifestyles [2,10,11]. Particular strains of *P*. *syringae* are especially pathogenic on specific host species [12–15], and can cause extensive damage to crop populations [12,16]. Several of these strains have been classified as pathovars, or lineages with specific unifying pathogenicity characteristics that specialize them on specific hosts. Previous genome comparisons of host-specific *P*. *syringae* strains showed that over 30% of genes within a strain’s genome are either unique to a single strain or are rare among other strains [7]. To date, however, only a few dozen of the thousands of variable genes have been shown to be adaptive in an environmentally specific manner [7,17,18].

*P*. *syringae* not only infects crop plant populations but also non-agricultural plant populations, including those of *A*. *thaliana*. Indeed, *P*. *syringae* is among the most abundant bacterial pathogens in *A*. *thaliana* leaves [19,20], causing reductions in fitness upon infection [21]. Interestingly, the evolution of *P*. *syringae* in *A*. *thaliana* populations appears to differ from that of *P*. *syringae* in crop populations. While crop-infecting strains frequently exhibit clonal, genetically monomorphic expansions and obvious disease symptoms [12,14,22], those *P*. *syringae* isolated from *A*. *thaliana* to date exhibit less obvious symptoms and more extensive genetic diversity [23], a diversity that is maintained even at a regional scale. The reason for this contrast in pathogenic genetic diversity between crops and a non-agricultural plant is unknown (though it is easy to speculate [24]). What is evident is that *P*. *syringae* colonizes *A*. *thaliana* populations successfully and frequently, and reduces yield [19,21,25].

The success and abundance of *P*. *syringae* in *A*. *thaliana* provides the opportunity to determine the evolution of *P*. *syringae* gene content within a single host species (*A*. *thaliana*) and to contrast this diversity with that observed among host species. Here, we begin to characterize gene content variability by genotyping 76 strains of *P*. *syringae* that reside on the same host species in the same geographical region of the Midwestern United States, and then fully sequencing 18 of these strains. We find that strains of *P*. *syringae* collected from *A*. *thaliana*, some from the same host population, exhibit variation in gene content similar to that observed between *P*. *syringae* strains collected from different crop host species. At the same time, the *P*. *syringae* strains collected from *A*. *thaliana* uniquely share a genomic island that encodes toxins active against a broad range of microbes, raising the possibility that these strains gain an advantage by suppressing other microbes. Combined, our findings reveal both extensive genetic turnover and a conserved genomic island that suggests the importance of microbe-microbe interactions in the evolution of a pathogen in natural plant populations.

## Results

### A specific lineage of *P*. *syringae* infects *A*. *thaliana*

**Fig 1:**
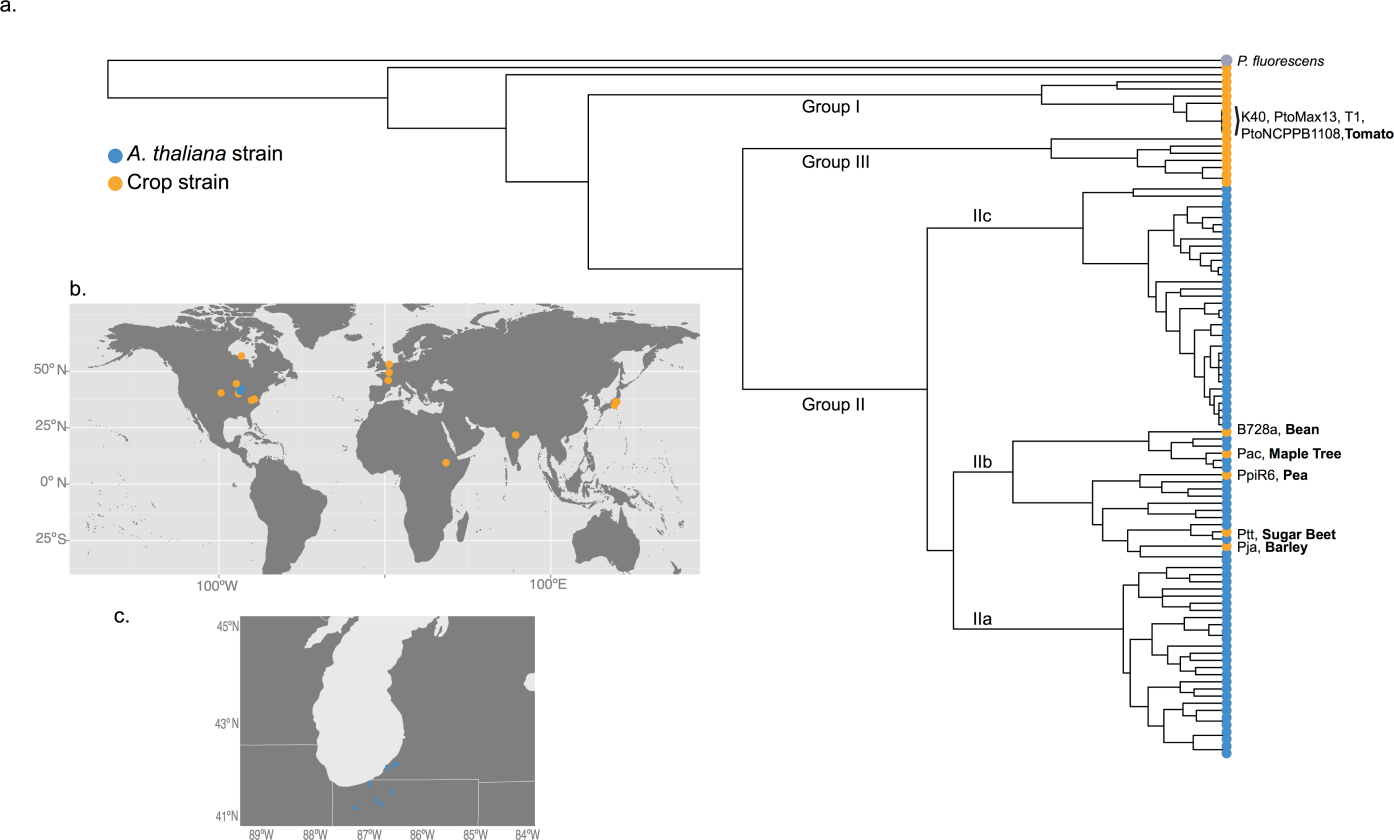
Composition of *P*. *syringae* in *A*. *thaliana* in the Midwestern USA. (a) Majority rule consensus tree based on six MLSA loci in *P*. *syringae*. Blue circles represent *P*. *syringae* strains collected from *A*. *thaliana* plants. Orange circles represent strains collected from crop plants. The names of select crop strains are provided next to the corresponding node along with the host of isolation in bold. The *A*. *thaliana* strains are restricted to group II while the crop strains span the *P*. *syringae* phylogenetic tree. (b) & (c) Geographic distribution of strains whose genomes were analyzed in this study.

**Fig 2:**
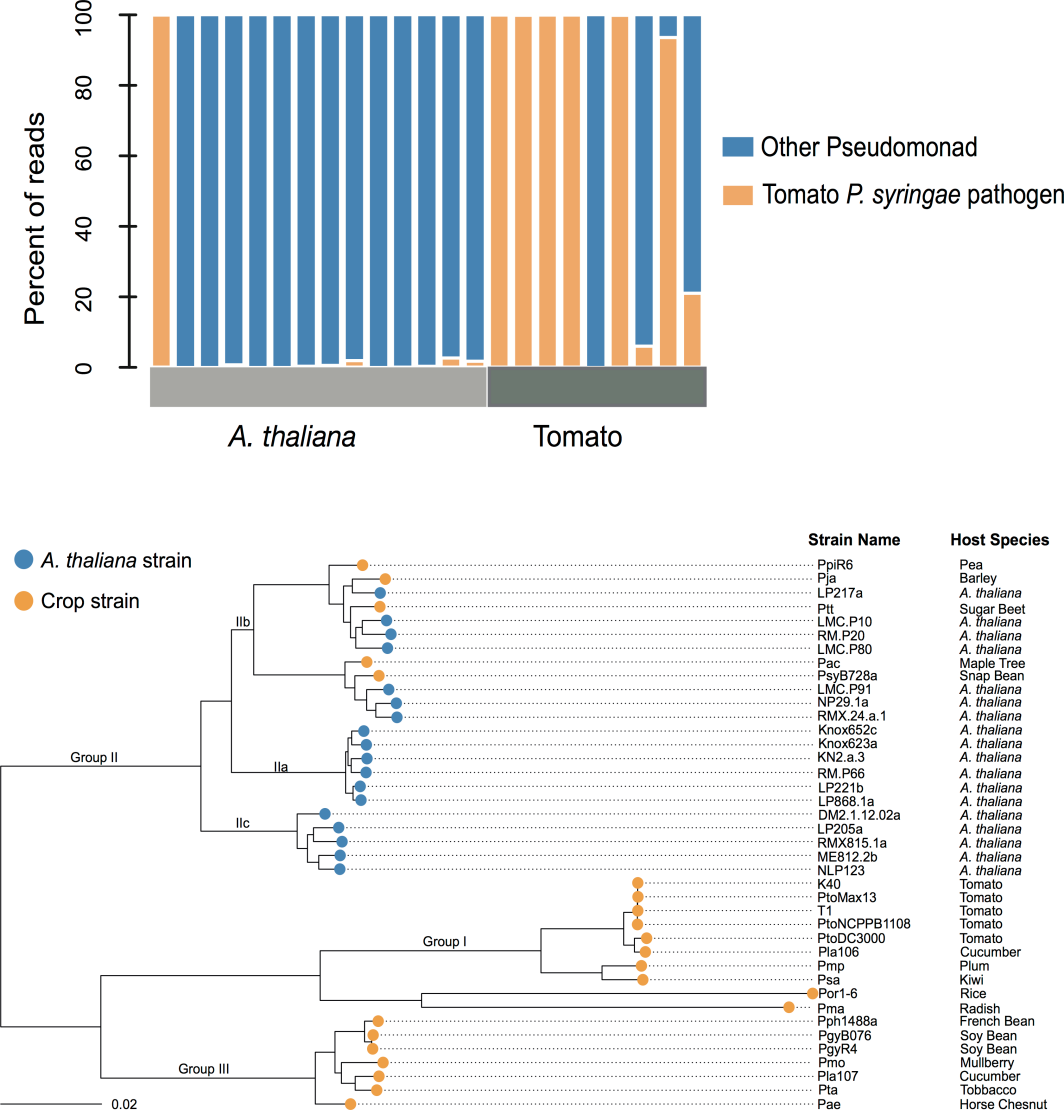
*P*. *syringae* from *A*. *thaliana* are genetically diverse, but are derived from one phylogenetic clade. (a) Pseudomonad composition of leaves of *A*. *thaliana* and tomato collected from the Midwestern USA was assessed via phylotyping of *gyrb*. The composition of Pseudomonads was significantly different between the leaves of tomato and *A*. *thaliana* (P = 0.008, Wilcoxon-rank-sum test), with the tomato leaves composed primarily of strains resembling the abundant tomato strains. (b) Maximum likelihood phylogeny of strains sequenced in this study based on 492 conserved genes. The scale bar indicates 2% sequence divergence. *A*. *thaliana* strains were primarily derived from phylogenetic group II, a result recapitulated in the wider MLSA (Fig 1). Group IIc lacks a canonical T3SS [27].

To study the gene content diversity of *P*. *syringae* in Midwestern USA populations of *A*. *thaliana*, we first sought to characterize *P*. *syringae* phylogenetic diversity in this area. *P*. *syringae* is both abundant and pathogenic within these *A*. *thaliana* populations, inducing a fitness cost in infected individuals of up to 25% [19,21]. We first determined the phylogenetic distribution of the *P*. *syringae* that colonize *A*. *thaliana* by performing Multilocus sequencing analysis (MLSA) of six loci [26] in 99 strains. The MLSA indicated that *P*. *syringae* isolates from *A*. *thaliana* cluster nearly exclusively within one specific lineage of *P*. *syringae* (group II as represented in Fig 1A, despite the fact that pathogenic *P*. *syringae* is abundant in several other phylogenetic lineages). Note, however, that those lineages collected from *A*. *thaliana* are not genetically identical, and instead, several crop strains from phylogenetic group II are more closely related to several *A*. *thaliana* lineages than are other *A*. *thaliana* lineages (Figs 1A and 2B).

While this result suggests the preferential colonization of group II in *A*. *thaliana*, it is also possible that the skewed phylogenetic distribution is the result of population structure of *P*. *syringae* within the Midwestern USA. However, previous studies reveal the genetic diversity of *P*. *syringae* in the Midwest [28,29] to be higher than that maintained on *A*. *thaliana* [23]. To explicitly test whether *P*. *syringae* in the Midwestern USA are from only this one lineage, we sampled *Pseudomonas* from both *A*. *thaliana* populations and nearby agricultural tomato crops in the Midwestern USA in the fall of 2013. Through high-throughput genotyping of the *gyrb* gene, we confirmed that group II [7,26,30] dominates within *A*. *thaliana* (Fig 1) yet another clade dominates within tomato crops (Fig 2A). The samples were collected within two weeks of one another, reducing the probability that differences in composition are due to temporal changes in microbial communities. These results suggest that a specific subset of *P*. *syringae* belonging to group II preferentially proliferates in *A*. *thaliana* populations.

### *P*. *syringae* on *A*. *thaliana* exhibit variation in gene content

**Fig 3:**
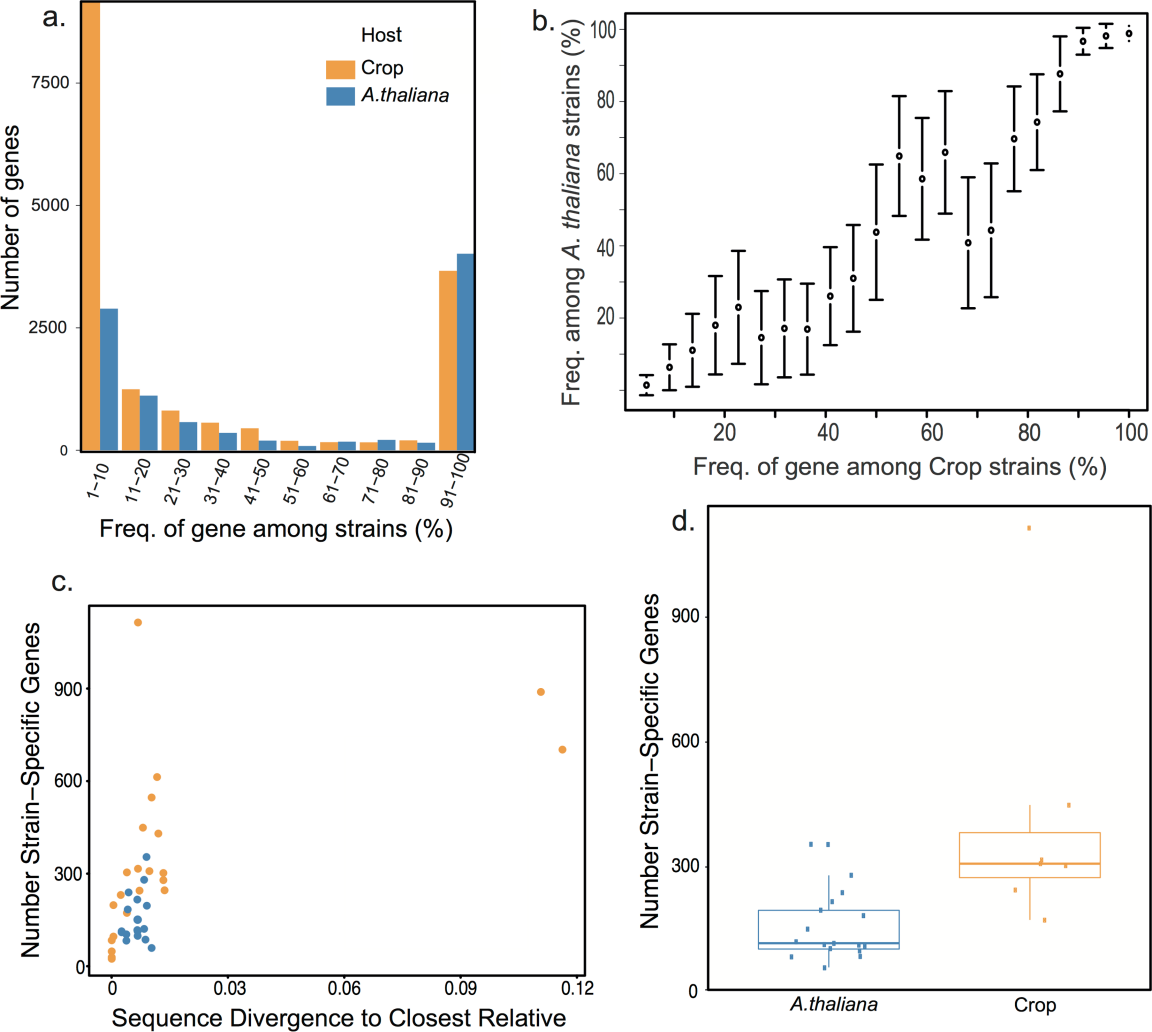
Extensive gene content variation in *A*. *thaliana* strains mirrors variation between strains from different hosts. (a) The frequency of genes among the 18 *A*. *thaliana* strains was compared to the frequency of genes across 22 strains collected from different host species, in different locations. Values were binned at 0.1 frequency increments with the number on the x-axis denoting the frequency of a gene across strains. *A*. *thaliana* and crop strains have a similar number of fixed and high frequency genes, suggesting that both groups have similar numbers of genes important for survival. However, crop strains have significantly more singleton genes (Welch’s t-test, P = 0.003). (b) Correlation between the frequency of a gene among crop strains and the frequency of the gene among *A*. *thaliana* strains. Results are shown as the mean +/ the standard error. (c) Correlation between sequence divergence between a strain and its closest relative with the number of strain-specific genes in that strain (d) The effect observed in (c) is significant also when comparing only those crops strains with divergences similar to those of *A*. *thaliana* strains (P = 0.003, Wilcoxon-rank-sum test). Results are presented as a box plot, with the mean, 5,25,75 and 95^th^ percentiles illustrated.

To assess gene content diversity in *A*. *thaliana P*. *syringae* strains, we sequenced the genomes of 18 randomly selected strains of *P*. *syringae* on *A*. *thaliana* [25] (Fig 2B) and compared them to the genomes of 22 largely host-specific crop strains from diverse geographical locations (Fig 1B and 1C). Previous genome comparisons of *P*. *syringae* isolated from diverse crop species found that more than 30% of genes within a genome are unique or rare among crop strains [7]. In this study, we re-annotated all genomes for consistency of annotation methodology between studies and relaxed the definitions of conservation to accommodate differences in assembly qualities between strains and studies. We also verified that differences in assembly quality did not significantly influence the number of genes annotated per genome (S1 Fig). Our findings support the previous findings of diversity, observing that an average of 32% of the genes within a crop strain’s genome (1710/5378 genes) varies in presence across crop strains. Although such large-scale differences in gene content have frequently been thought to result from host-specific adaptations [31] our analysis also revealed high levels of gene content variation among *A*. *thaliana* strains (Fig 3A). An analysis of the gene-frequency-spectrum of *P*. *syringae* within *A*. *thaliana* host populations revealed that the frequency distribution of genes among *A*. *thaliana* isolates closely resembles the gene-frequency-spectrum of *P*. *syringae* across diverse crop hosts: a similar number of genes are conserved across strains and a similar number of genes are distributed at intermediate frequency. An average of 79% of the genome of an *A*. *thaliana* strain is conserved (found in more than 90% of strains) across *A*. *thaliana* isolated strains (4014/5098 genes). Stated differently, 21% of an *A*. *thaliana* strain genome on average is not conserved across *A*. *thaliana* strains in comparison to the 32% that is not conserved across crop strains.

### Crop strains encode more strain-specific genes

Perhaps the most prominent observable difference between the frequency spectrum of genes between *A*. *thaliana* and crop stains is that the pan genome of the crop strains contains an increased number of singleton genes (roughly three times more strain specific genes per crop strain with an average of 311 vs. 110 strain specific genes per crop and *A*. *thaliana* strain respectively), present in only a single sequenced strain (Fig 3A). Such increased numbers of singletons could reflect host-specific adaptations (but see [8] and discussion in Methods section for alternatives). An alternative hypothesis for this excess of singletons is the greater phylogenetic distance among crop strains. It has been shown that gene content differences frequently correlate with phylogenetic relatedness [32]. Sampling of more closely related strains (such as *A*. *thaliana* strains) may result in deeper sampling of rare genes making the identification of singletons less likely. To explore this possibility, we tested the relationship between nucleotide divergence between strains and the number of singletons observed (Fig 3C and 3D). When we considered only those crop strains that were as closely related to other strains as are the *A*. *thaliana* strains, we still found a significantly higher number of strain-specific genes per crop strain (Fig 3D) (Wilcoxon-rank-sum test, P = 0.003). These results suggest that the increased number of singletons in crop strains is not fully explained by phylogenetic distance.

Whether most singletons are functional and, furthermore, whether they are adaptive is unclear. We could not detect any functional differences between the genes that exist as singletons in our two populations: in a comparison of gene functions for these singletons, we found significant enrichment only for genes of unknown function (Fisher’s exact test q-value<0.01, [33]) but not for any other functional category.

### The overlap of pan genomes between and within hosts

When considering particular genes that are variable in their presence, we find the frequency of a gene’s presence in strains collected from different hosts to be a good predictor of its frequency in co-occurring strains in *A*. *thaliana* populations (Fig 3B; Pearson correlation r = 0.94, P < 0.001). That is, the same genes are variable within and between host species. This result suggests that the molecular and/or evolutionary processes that generate and maintain presence/absence polymorphisms are recapitulated within and between hosts and geographic regions. This result is robust to the exclusion of core genes (Pearson correlation r = 0.50, P < 0.001).

### The similarity of the within and between host core genomes

The core genome of a microbial taxon group, defined as those genes conserved across isolates of that group, is comprised primarily of genes that have been vertically inherited. The core genome is therefore thought to be enriched for genes essential for survival [34]. The core genome of *A*. *thaliana* and crop strains is similar in gene number (4014 genes vs. 3665 genes respectively, defining core genes to be those that are present in >90% of strains within each group). Furthermore, largely the same genes are found in the core genomes of the *A*. *thaliana* and crop strains of *P*. *syringae*; 88% (3551 genes) of the core for *A*. *thaliana* strains overlap with the core for all crop strains. This suggests that the majority of genes fundamental to survival within the relatively constrained Midwestern USA *A*. *thaliana* environment are the same as those required for survival across multiple host species and geographic locations.

### A genomic island encoding toxins active against plants and microbes is enriched in *A*. *thaliana* strains

While patterns of gene-content variation are similar between strains associated with the same and different hosts, 436 genes are specifically conserved among *A*. *thaliana* strains (and vary in presence across crop strains). This host-specific conservation could be the result of host specific adaptive conservation. Comparison of the gene functions enriched in the *A*. *thaliana*-specific suite of genes to those genes conserved across all strains reveals substantial enrichment for functions associated with phytotoxin production (S2 Fig) (322-fold enrichment, Fisher’s exact test q-value<0.001 [33]) and minor enrichment for transcriptional regulation (2-fold, Fisher’s exact test q-value<0.001). Included in the enriched genes associated with phytotoxin production are those encoding the biosynthetic pathway for syringomycin and syringopeptin, toxins which exhibit broad host-range plant virulence and antifungal properties [35]. Numerous studies have demonstrated that syringomycin and related non-ribosomal lipodepsipeptides can suppress other microbes and increase growth of the pathogen within agricultural plant species [35,36].

**Fig 4:**
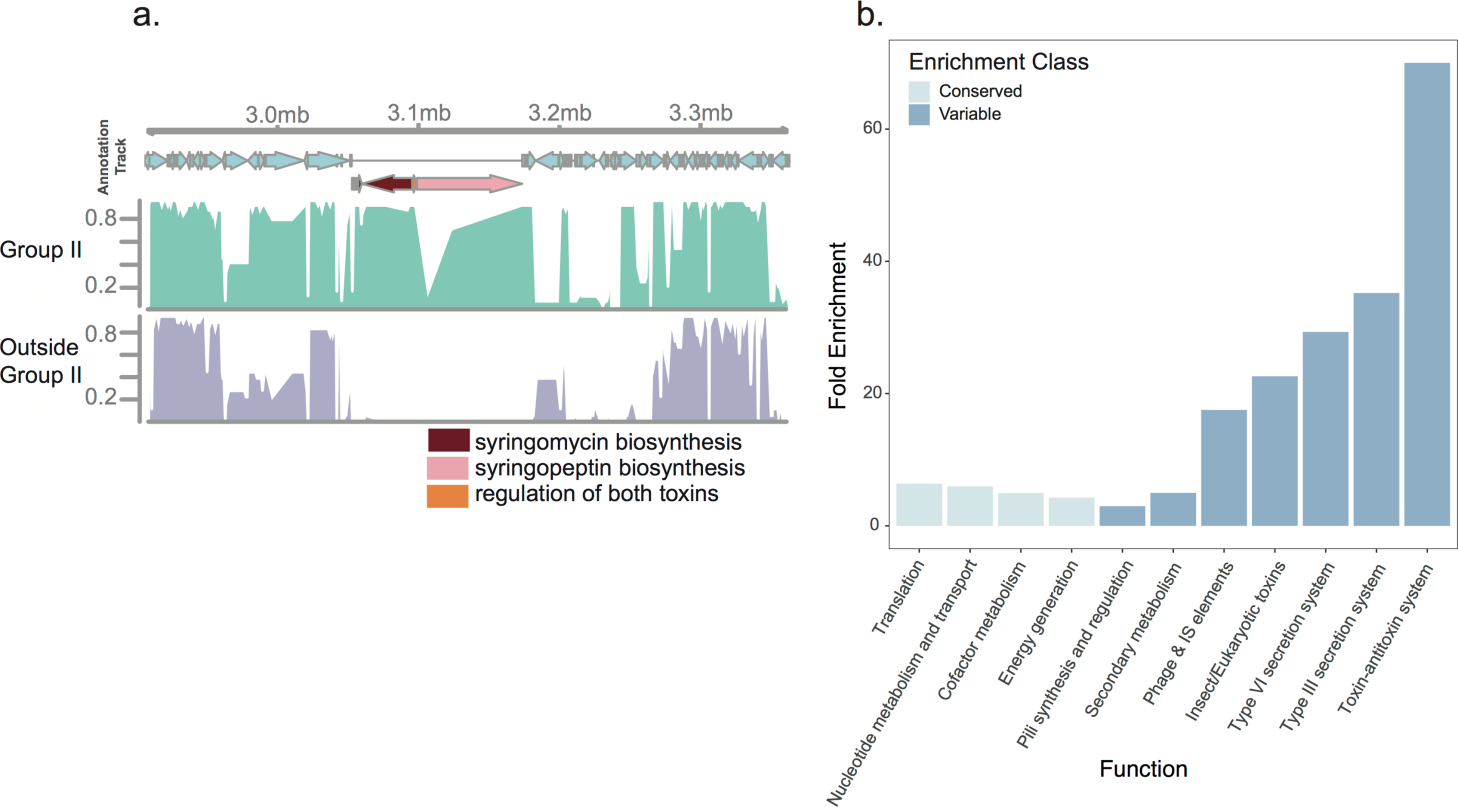
Genes involved in biotic interactions evolve quickly amongst *A*. *thaliana* strains. The genomes of *P*.*syringae* from *A*. *thaliana* contain genes whose products have the ability to suppress a diversity of microbes. (a) A view of gene content conservation surrounding the syringomycin-syringopeptin genomic island. The x-axis denotes the position of the gene in the *P*. *syringae* strain B728a. The y-axis shows the percent conservation across crop and *A*. *thaliana P*. *syringae*. A genomic island encoding the syringomycin biosynthetic cluster is conserved across strains of *P*. *syringae* from phylogenetic group II. The genes responsible for synthesis of syringomycin and syringopeptin are colored cranberry and pink, respectively. The genes annotated within the orange arrow are involved in the regulation of both toxins. (b) Fold enrichment of gene functions in the conserved and variable portions of the *P*. *syringae* genome. Only gene categories that were significantly enriched in either the conserved or variable gene sets were included in this figure. Significance was assessed via Fisher’s exact test, and a false discovery rate significance level of 0.01.

The relevance of syringomycin-syringopeptin biosynthesis to *A*. *thaliana*-associated success is further supported by the phylogenetic distribution of toxin-associated genes. Because *A*. *thaliana* isolates derive almost exclusively from a single phylogenetic clade, phylogenetic group II (Figs 1 and 2), we aimed to identify genomic regions specific to this clade. We filtered gene content for those genes that were both unique to, and at high frequency (>90%) within, phylogenetic group II. Fifty-six genes met these criteria (Fig 4A, S2 Table), 29.0% (16/56) of which lie in the gene cluster that encodes the proteins necessary for syringomycin-syringopeptin biosynthesis (Fig 4A) [17,35]. Indeed, functional tests of phylogenetic group II strains have confirmed their fungal-suppressive capacity [26]. The remaining phylogenetic group II specific genes are distributed throughout the genome (S2 Table).

Both the functional enrichments and the phylogenetic distribution reveal the tight correlation between strain abundance in *A*. *thaliana* and the presence of the syringomycin-syringopeptin biosynthetic cluster. Future functional work should investigate whether these broad host-range toxins are necessary for *P*. *syringae* persistence in *A*. *thaliana* populations as well as natural plant populations more generally.

### *A*. *thaliana*-associated *P*. *syringae* encode few effectors

The Type-III secretion system (T3SS) and its related effectors are prime *a priori* candidates for involvement in adaptation to different host environments. Effectors and the T3SS are central to the capacity of *P*. *syringae* to infect plants, and the presence or absence of specific effectors can dictate the success of an infection [37]. Effector composition differs between crop strains, and is not tightly correlated with phylogeny, suggesting the rapid gain and loss of these genes [7]. Through computational annotation of the effector content in our *P*. *syringae* strains, we found that the effector composition of *A*. *thaliana*-associated *P*. *syringae* strains also varied, with each strain containing homologous sequence to an average of 6.8 effectors. Note that we consider all homologous sequences (50% identity over 50% of the length), including effectors that may also be truncated. The crop *P*. *syringae* genomes contain an average number of effectors more than four times higher (29.4) than the stains associated with *A*. *thaliana*. Five of the sequenced *A*. *thaliana*-associated strains lack a canonical T3SS entirely and encode 0–1 effectors, with only hopAH2 found in these strains (S3 Fig). Note that the classification of hopAH2 as an effector has recently been questioned, due to the absence of the hrp box, or the N-terminus of the T3SS translocator.

The dearth of effectors in the phylogenetic group II that encompasses *A*. *thaliana* strains was previously noted [7], and postulated to be the result of a physiological trade-off between effector and toxin- mediated interactions with the host [38]. The *A*. *thaliana*-associated strains may, however, encode other effectors that have yet to be identified in *P*. *syringae*.

### Genes involved in biotic interactions are more likely to exhibit presence/absence polymorphisms

Many of the genes at the interface of host-pathogen interactions evolve quickly, exhibiting both presence/absence and nucleotide variability [7,26]. We determined the gene ontology classifications most frequently found in the variable vs. core genomes for the *A*. *thaliana P*. *syringae* sequences. As expected, the core genome is significantly enriched for basic cellular functions such as cell division and translation (Fig 4B). In contrast, the variable genome exhibits more than a 60-fold enrichment of genes involved in specific plant-microbe interactions (e.g., T3SS and pilus) and genes involved in microbe-microbe interactions (the type six secretion system) (Fig 4B). Virulence factors such as the T3SS and its myriad effectors frequently cluster in genomic islands that exhibit presence/absence polymorphisms (S4 Fig). Phage and insertion sequence (IS) elements are also enriched in the variable component of the genome (Fig 4B). These results indicate that those genes involved in biotic interactions exhibit among the highest rates of gain and loss and also reflect the clade-specific distribution of the syringomycin-syringopeptin synthesis. Whether this increased rate of acquisition and loss is the result of selection, or the result of particular molecular mechanisms (such as heightened rates of uptake and excisions [39,40]) is currently unknown.

## Discussion

Horizontal gene transfer and gene loss enable bacterial species to evolve rapidly. A consequence of this genetic malleability is that different strains of the same species can quickly become divergent in the genes they encode. Numerous studies have demonstrated that strains that have likely diverged phenotypically (such as those collected from different hosts) differ extensively in gene content. Whether this variation in gene content is the result of genome-wide adaptation to the colonization of specific environments remains a largely unanswered question. A key to identifying environment-specific genetic adaptations is determining gene content variation both within and between environments.

Here we characterized the extent of gene content diversity present in co-occurring strains of the ubiquitous plant pathogen *P*. *syringae*. Our comparison of whole genome sequences of *P*. *syringae* resident on a single host, *A*. *thaliana*, has revealed patterns of gene content variation very similar to patterns found for crop strains that specialize on diverse host species from diverse geographic locations. Specifically, gene content variation of strains collected from *A*. *thaliana* is 2/3 that of the variation of strains collected from disparate hosts. This variable portion of the genome consists of genes strongly enriched for microbe-host and microbe-microbe interactions. This raises the possibility that the majority of the variable genome may not be the result of adaptation to alternative hosts but, rather, a more general armament to survive in dynamic and variable host environments. The excess of strain-specific genes among crop strains is an intriguing observation, albeit with several possible explanations. Future work should characterize the function of candidate strain-specific genes in *in planta* survival.

There are also several plausible explanations for the maintenance of gene content diversity within co-occurring strains of *P*. *syringae*. *A*. *thaliana* is an annual weedy species, found at relatively low densities among several other plant species [41]. *P*. *syringae* that propagate in *A*. *thaliana* can do so only for a portion of the year (due to plant senescence), and must then disperse to other host species or non-host environments [2,25]. This variation in host occupation is likely to differ from the strains isolated from crops, several of which have evolved to specialize on their host of isolation [12,24,42]. The strains isolated from crops have repeatedly if not continuously infected crop host populations [12]

While *P*. *syringae* on *A*. *thaliana* may propagate in hosts other than *A*. *thaliana*, there is no evidence that these *P*. *syringae* predictably colonizes any other host. The extensive diversity of *P*. *syringae* strains in non-host environments suggests that many *P*. *syringae* opportunistically colonize a diversity of environments. It is also likely that the “*A*. *thaliana”* strains colonize other hosts, though at present we do not have evidence to support this. The different composition of strains infecting *A*. *thaliana* could be the result of differences in selective pressures in alternative non-host and host environments.

Another possibility is that genetic diversity observed between *A*. *thaliana* strains is a consequence of intraspecific differences in *A*. *thaliana* plants. The genes underlying the detection and response to microbes are among the most variable in *A*. *thaliana* populations [43], and differences in resistance traits could drive the diversification of pathogen genomes. Intraspecific differences in the host environment extend beyond host genetics. *A*. *thaliana* leaves and roots harbour thousands of microbial species [44–46]. These microbial communities differ between plants of the same genotype, plants of different genotypes and plants in different soils. Consequently, plant-associated *P*. *syringae* evolve both in response to interactions with the host, but also in response to interactions with other microbial species. The rapid turnover of genes involved in interactions with other microbes (such as the secretion systems) suggests that microbe-microbe interactions contribute to gene content variation within host populations.

It is important to consider the possibility that we observe similar levels of variation among *A*. *thaliana* strains and among strains collected from various crop hosts because novel genes do not drive adaptation to particular host environments [32,47–49]. That is, it is possible that there exists a pool of genes that will be more readily lost independent of environment, because their maintenance is not strongly favored in any environment [49]. It is also possible that there are genes that, across environments, are more readily acquired via HGT. This, in turn, may lead to the observed pattern by which most genes will be found at similar frequencies within and between environments. Indeed, our finding that the frequency of genes tends to be similar within and between hosts highlights the caution that should be taken when attributing the absence or presence of specific genes to the occupation of specific environments.

Despite the extensive gene content variation we observe among strains residing within *A*. *thaliana*, these resident strains of *P*. *syringae* are nevertheless characterized by the presence of a genomic island that encodes for the production of two toxins, syringomycin and syringopeptin. While the full breadth of their toxicities is not well characterized, they are effective against fungi, plants, and gram positive bacteria, pointing towards a potential ability of the *A*. *thaliana P*. *syringae* strains to suppress and outcompete other microbes and to infect diverse plant host species.

*P*. *syringae* in Midwestern populations of *A*. *thaliana* do not show evidence of specific adaptation to *A*. *thaliana* and instead exhibit features suggestive of a generalist lifestyle [50]. Perhaps as a consequence of this generalist lifestyle, the *A*. *thaliana P*. *syringae* employ novel tactics to promote their success. The lack of a T3SS in one third of the *P*. *syringae* that colonize *A*. *thaliana* allows them to avoid detection, although these strains pay a penalty in terms of virulence when T3SS+ pathogens are not present [23]. Indeed, in a broad GWAS analysis of the host factors shaping microbial communities, there is only a modest contribution from plant *R* genes, the genes classically suspected of engaging in arms races [45]. Clearly, an understanding of microbe-microbe interactions is essential for understanding the structure and distribution of microbial communities *in planta*, as well as the spread of disease.

## Materials and methods

### Description of strains

*P*. *syringae* strains genotyped and sequenced in this study were originally isolated from *A*. *thaliana* populations in the Midwestern USA between 2000 and 2014. The 18 *P*. *syringae* strains that underwent full genome sequencing were isolated from nine *A*. *thaliana* populations residing in agricultural fields in Northwestern Indiana and Southwestern Michigan, USA. These populations are separated by an average distance of 28km and a maximum distance of 98km. The 22 crop strains were isolated from 22 distinct locations, separated by an average distance of 9648km (Fig 1, S1 Table). The 18 strains isolated from *A*. *thaliana* span the genetic diversity of strains from the *A*. *thaliana* environment, encompassing groups IIa-c [25].

### Genomic DNA extraction, DNA sequencing, assembly and annotation

Total DNA was extracted using the Puregene (Illumina) extraction kit from a single colony that was picked and grown overnight in 5mL of King’s B media. Colonies were diluted 1:10 in the morning after 12-16hrs, and grown 2–6 hours to an OD600>0.1, which was then followed by DNA extraction. Mate-pair libraries were constructed at Argonne National laboratories and paired end libraries constructed at Beijing Institute for Genomics.

100-base pair reads were generated from the sequencing of mate-pair libraries for 18 *A*. *thaliana* strains on a Genome Analyzer II (Illumina). 75-bp un-paired reads were also generated for each of the strains. *De novo* assembly was performed using Velvet 1.1.05 [51]. Minimus2 from the Amos package [52] was used to merge contigs generated from the different sequencing methods. The genomes were annotated with Rapid Annotation using Subsystem Technology (RAST) server [53]. The 22 previously annotated crop strains were re-annotated using the RAST server for consistency in annotation between all strains. The draft genomes used in this study consisted of single up to thousands of contigs. Due to unavoidable errors in contig assembly, multiple contigs may overlap the same genomic region, resulting in the duplication of genomic regions in an assembly. In consideration of this possibility, duplicate genes (100% identity) within a genome were removed from the annotated dataset, and not considered in the analyses. The genome assembly statistics are presented in Table 1.

**Table 1:** Assembly information for strains analyzed. The N50 for the strains sequenced in this study is for unscaffolded contigs. Several of the previously sequenced assembled genomes are scaffolded, however, increasing the N50 for these genomes.

Strain	Gene Num.	Num. Contigs	Size (bp)	N50
Pph1448a	5454	3	6112448	Complete
PsyB728a	5254	1	6093698	Complete
PtoDC3000	5661	3	6538260	Complete
PtoMax13	5525	349	6105073	62407
PtoNCPPB1108	5482	304	6082048	47802
Pmp	5327	969	6039297	15161
Pla107	6248	791	6759945	22550
Pmo	5693	3414	6392728	5634
Pja	5674	4661	6380619	4021
PpiR6	5872	5099	6520586	3003
Pma	5474	878	6221751	17222
Psa	5245	941	5849032	14086
Pla106	5293	798	5895184	15738
Ptt	5262	3776	6243278	4753
Pac	5498	1179	6183769	12409
Pta	5541	1613	6158837	16098
PgyB076	5652	104	6236653	202511
PgyR4	5355	108	5905212	3723
Pae	5308	915	5960467	16806
T1	5587	122	6145942	150139
K40	5557	582	6153658	26013
Por1-6	5290	2855	6704257	10037
DM2.1.12.02a	5180	157	5914114	88439
LMC.P10	5226	172	6238204	83072
LMC.P80	5136	137	6189597	120774
LMC.P91	5374	243	6324900	59429
KN2.a.3	5201	133	5804868	120373
Knox623a	5391	554	5946364	22175
Knox652c	5071	160	5905380	95163
NL.P123	5030	220	5855665	61758
NP29.1a	5088	208	6088841	76982
LP217a	5242	382	6224113	47360
LP221b	4967	122	5939644	95628
LP868.1a	5153	123	5907756	111176
RM.P66	5290	70	7081112	284403
RMX.24.a.1	5367	224	6271973	69103
RM.P20	5153	159	6229891	99197
RMX815.1a	5077	167	5565846	67363
ME812.2b	4888	334	5636659	56761
LP205a	4964	478	5698429	30345

### Phylogeny construction

To determine the phylogenetic relationship between the strains isolated from *A*. *thaliana* we performed multi-locus-sequencing-analysis (MLSA). The MLSA-based phylogeny evaluating the relationship between 76 strains of *P*. *syringae* isolated from *A*. *thaliana*, 22 crop strains, and a *Pseudomonas fluorescens* outgroup was constructed from six housekeeping genes [54] *cts*, *cgi*, *gyrb*, *can*, *gap A* and *rpoD* using ClonalFrame, a software optimized for estimating phylogenies in the face of bacterial recombination. ClonalFrame [55] was run for 10000 burn-in iterations, 10000 post-burn in iterations, with sampling on every 10th generation. The phylogeny presented represents the 50% majority rule consensus tree. To corroborate the division on the tree, we also constructed a maximum-likelihood phylogeny from the concatenated MLSA sequences. This phylogeny supported the restriction of *A*. *thaliana* strains to phylogenetic group II. The phylogeny for the 40 genomes used for calculations of sequence divergence in this study was constructed using dnaml [56]. Two hundred sixty four genes that were reciprocal best hits were concatenated and used in the dnaml analysis. Orthologs used for phylogenetic analyses were defined as those genes that were reciprocal best hits, and aligned across 100% of the sequence in a global alignment comparison using fasta36 [57]. We use this stringent criterion for determining orthologs when estimating the species phylogeny because we aim to minimize the possible effects of horizontal gene transfer (HGT) on the phylogenetic tree we generate. Highly conserved genes, both in presence and in sequence length are more likely to be vertically inherited than those genes that vary between strains. The 436 genes that met the described criteria and that were observed in all 40 *P*. *syringae* strains and the outgroup *P*. *fluorescens* Pfo-1 strain were concatenated for each strain, and a maximum-likelihood tree for concatenated genes was determined using dnaml [56,58]. While it is possible that some of these 436 conserved genes have been subject to HGT in some of the studied strains, the signal these relatively rare HGT events introduce should be weak considering most concatenated genes are likely to have been inherited vertically along the tree.

### Determining Pseudomonad composition of Midwestern tomato and *A*. *thaliana* leaves

Leaves from tomato and *A*. *thaliana* in agricultural and natural populations respectively were collected in November 2013. Single leaves were removed from tomato plants irrespective of plant disease state and frozen immediately in liquid Nitrogen. Whole *A*. *thaliana* rosettes were collected, and frozen immediately in liquid Nitrogen. *A*. *thaliana* roots were removed from the rosette after which *A*. *thaliana* and tomato leaves were processed identically. The plant material was lyophilized overnight until complete dehydration. The powerplant pro DNA extraction was then used to extract DNA from the lyophilized material. Two sequential extractions were performed on the tomato tissue to remove residual secondary metabolite contamination, which inhibited subsequent polymerase-chain reactions. Bar-coded PCR amplification of the different extractions was then performed to amplify a fragment of *gyrase b* using primers modified from [26] to be able to anneal to an Illumina flow cell. The barcoded samples were then sequenced on the MiSeq using a 500 cycle kit. Fifty samples, 25 from *A*. *thaliana* and 25 from tomato produced viable reads mapping to *gyrase b*. The resulting reads were then clustered with the usearch-global algorithm to a library of *gyrase b* sequences generated from sequences within the PAMDB [59]. Those clusters that mapped with 75% identity were considered for further analysis.

### Identification of conserved vs. variable genes

The pan genome of *A*. *thaliana* strains and all 40 strains for which full genome sequence data were available were determined using a method similar to that described in [7] but with slight amendments including the alignment algorithm used, and the parameters for determining homologs. In brief, we determined the presence and absence of genes within a genome using an iterative global alignment with the software fasta36 [57]. We compared the translated sequences using a cutoff of 50% homology over 50% the length of one of the two genes. Starting with the ORFs in Pph1448a as the initial pan genome, we compared the translated ORFs of each subsequent draft genome to the pan genome, and determined which genes in the draft genome had not been observed in the compiled pan genome. These unique genes were then added to the pan genome for the next iteration of sequence comparison with the next draft genome. After these iterations had been completed for all 40 strains, we compared the ORFs of each genome to the completed pan genome. This step was necessary to properly incorporate information for genes that were homologous to more than one gene in the pan genome. This method for characterizing the content of the pan genome scores a gene as present if homology is exhibited over either of the genes in a comparison, fragmented coding sequences will align with the intact ortholog, and these fragments will not inflate the number of genes in the pan genome as described [8].

### Calculation of sequence divergence

For the calculation of sequence divergence between two strains, we considered only those genes that were found in all *P*. *syringae* strains, that were reciprocal best hits, and that aligned across 100% of their sequence (fasta36 [57]). These 436 genes were concatenated for each strain and sequence divergence was calculated using the method of [56].

### Identification of effector repertoire

Annotations of previously identified *P*. *syringae* effectors were obtained from [7]. These annotations included 79 effectors, with several effectors represented with more than one allele. The presence of an effector in the *A*. *thaliana*- associated *P*. *syringae* genomes was assessed by using tblastn [60] to identify protein homologs in the genomes. A homolog was considered to be present if it matched the previous annotation with 50% identity at the protein level over 50% of the protein. Seven effectors are found in more than 50% of the group II strains. Three of these lie in the conserved effector locus cluster (*avrE*, *hopA1*, *hopM1*) and three lie in another operon (*hopAH1*, *hopAG1*, *hopAI1*). Interestingly, a homolog of *hopAH2* is found in every *P*. *syringae* genome (except RMX815.1a) in every phylogenetic clade, including the *P*. *syringae* strains that lack the canonical T3SS. *hopAH2* lacks a hrp box, and its status as an effector has come into question (http://www.pseudomonas-syringae.org/). It is important to note that in this study we identified only effectors that had previously been annotated, and that our results do not preclude the presence in these strains of effectors that have yet to be annotated as such.

### Determining functional enrichments

A recent study [61] manually annotated the gene functions of a *P*. *syringae* strain closely related to the strains we sequenced here, annotating genes as one of 63 functional categories. These annotations, while not covering the entire genome, are more specific than those provided by RAST and other automated annotation method. Two-thousand eight hundred and fifty genes were included in the enrichment annotations. We classified 2404 of these genes as conserved (present in at least 17 of the 18 genomes or 94.4% of the genomes), and 446 as variable. Using the 63 categories of annotation, we determined enrichments for functions within the conserved and variable categories. Statistical significance was assessed using fisher’s exact test, and a False Discovery Rate of 0.01 [33].

Comparisons of the functional enrichments of the strain specific genes were performed using the functional annotations of all genomes generated in the RAST pipeline [53]. Subsystem annotations were compared across genomes with the R package ‘TopGO’ (created by Adrian Alexa and Jorg Rahnenfuhrer), and significant enrichment was assessed using fisher’s exact test, and a False Discovery Rate of 0.01 [33].

## Supporting information

S1 Fig Relationship between genome assembly N50 and number of genes identified per genome. The relationship between genome assembly quality and the number of genes (non-duplicates) annotated per genome is not significantly different from zero (linear regression, P = 0.291). The opaque gray shows the 95% confidence interval for the predicted relationship.
(PDF)

S2 Fig Fold enrichment of gene functions enriched among genes conserved specifically in *A*. *thaliana* strains in comparison to genes conserved across all strains. Only gene categories that were significantly enriched in either the conserved or variable gene sets were included in this figure. Significance was assessed via Fisher’s exact test, and a false discovery rate of 0.01.
(PDF)

S3 Fig *A*. *thaliana* associated strains encode few canonical *P*. *syringae* effectors. The x-axis details the 79 effectors (or alleles of effectors) that were identified previously in *P*. *syringae* genomes [7]. The y-axis shows the 18 genomes sequenced in this study, and whether an orthologue of these effectors was identified (dark blue indicates presence in genome).
(PDF)

S4 Fig Gene content variation at the conserved effector locus. Conservation of genes along the conserved effector locus (shaded in red). Approximately one third of strains collected from *A*. *thaliana* lack the canonical T3SS [23].
(PDF)

S1 Table Location of isolation for strains analyzed in this study. Information was not available for several strains (listed as "NA"). When non-specific isolation information was provided by collector (such as country of origin), the latitude of country is provided. LMC is an abbreviation for Lake Michigan College.
(PDF)

S2 Table Genes unique to and conserved in group II. This table shows the position in the B728a genome of each of the genes unique to group II. Fifty-six genes were conserved in 90% or more of the strains in group II, but absent outside group II. 29% of these genes lie in the syringomycin/syringopeptin biosynthetic cluster, highlighted in Cyan.
(PDF)
