# Supplementary figures and images for: Similar levels of gene content variation observed for Pseudomonas syringae populations extracted from single and multiple host species
Source: PLoS One. 2017 Sep 7;12(9):e0184195. doi: 10.1371/journal.pone.0184195 (PMC5589212; doi:10.1371/journal.pone.0184195)

Figure S1

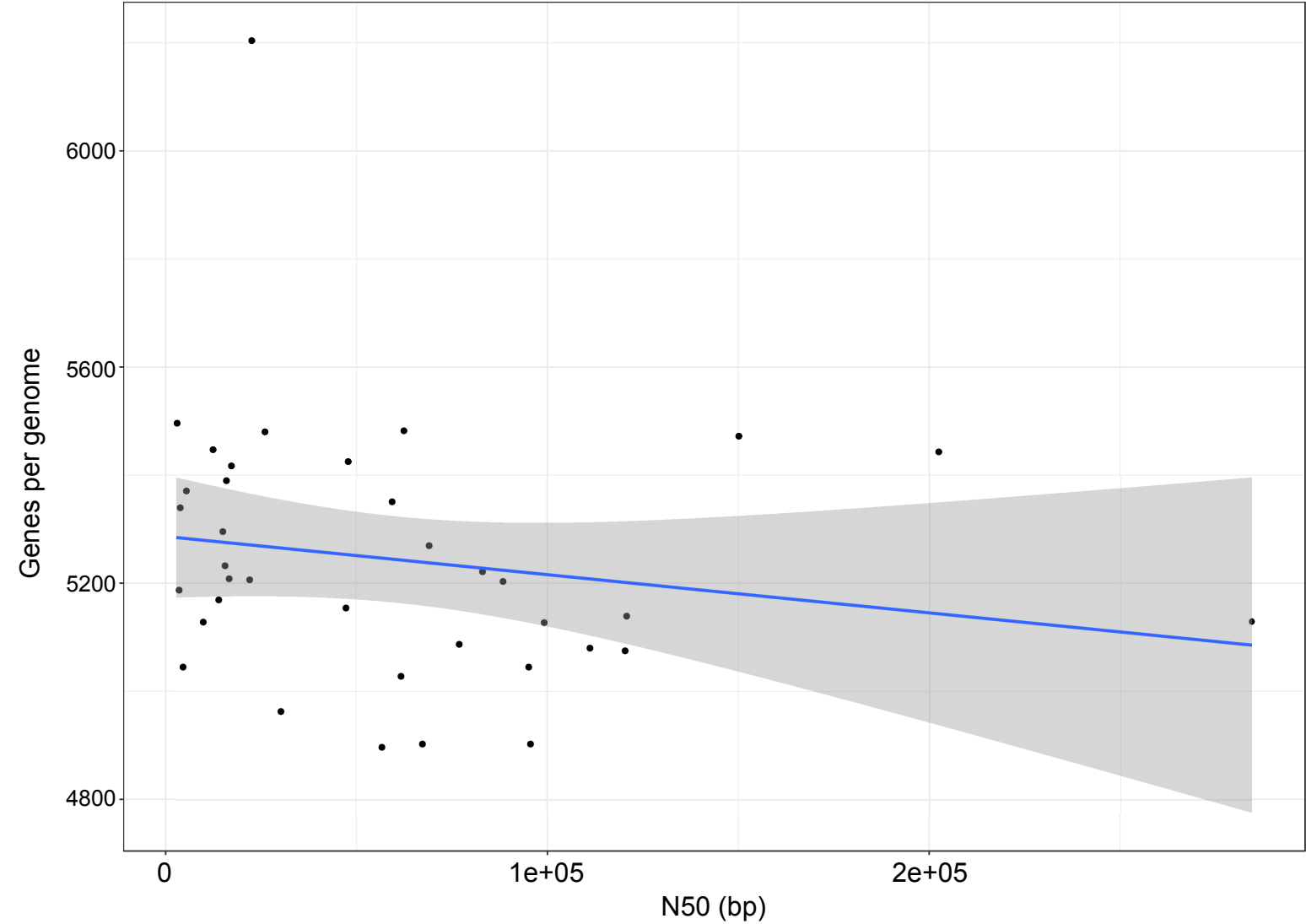

Supplement: S1 Fig — The relationship between genome assembly quality and the number of genes (non-duplicates) annotated per genome is not significantly different from zero (linear regression, P = 0.291). The opaque gray shows the 95% confidence interval for the predicted relationship. (PDF) [file pone.0184195.s001.pdf]

Figure S2

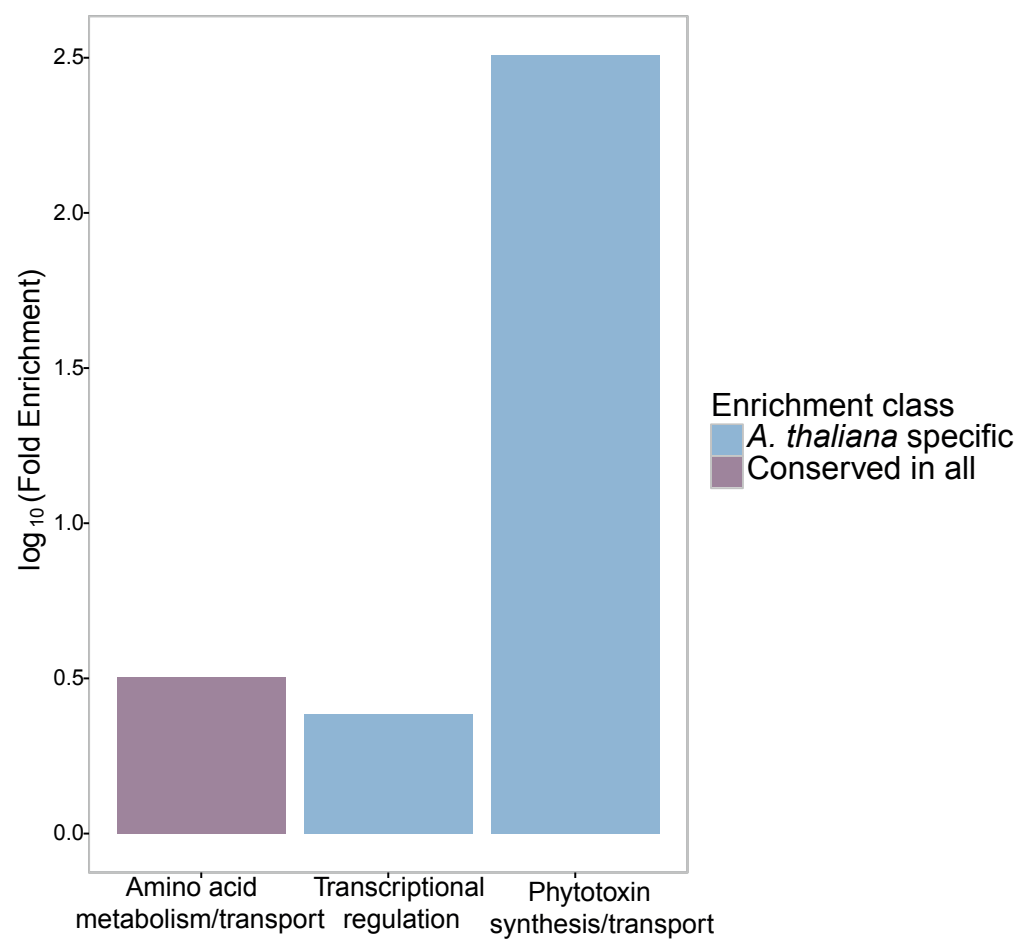

Supplement: S2 Fig — Only gene categories that were significantly enriched in either the conserved or variable gene sets were included in this figure. Significance was assessed via Fisher’s exact test, and a false discovery rate of 0.01. (PDF) [file pone.0184195.s002.pdf]

Fig. S3

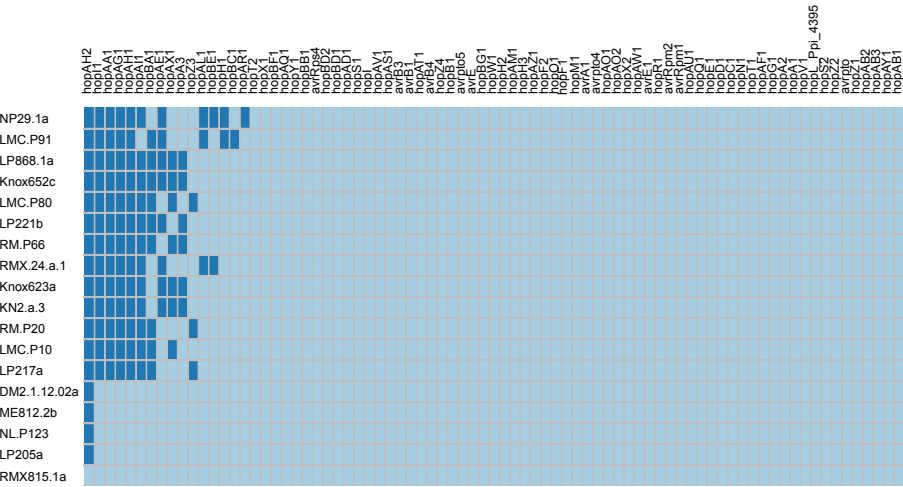

Supplement: S3 Fig — The x-axis details the 79 effectors (or alleles of effectors) that were identified previously in P. syringae genomes [7]. The y-axis shows the 18 genomes sequenced in this study, and whether an orthologue of these effectors was identified (dark blue indicates presence in genome). (PDF) [file pone.0184195.s003.pdf]

Fig. S4

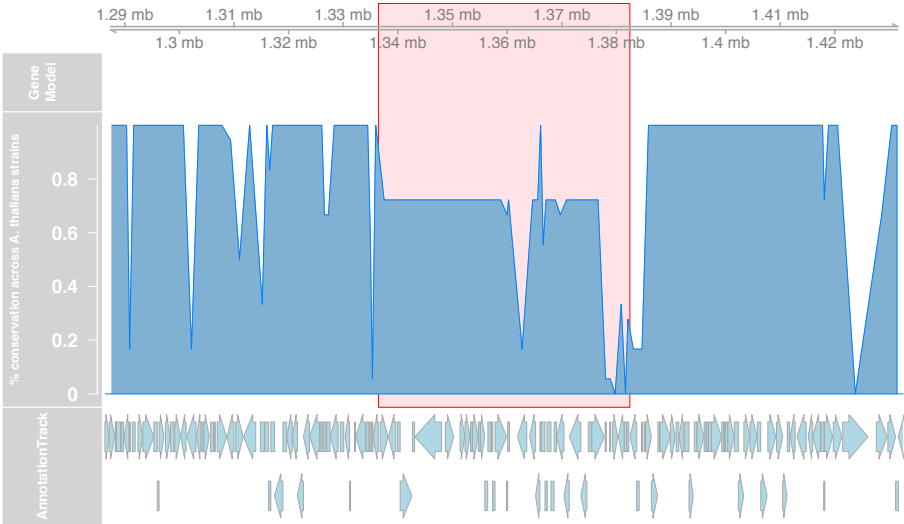

Supplement: S4 Fig — Conservation of genes along the conserved effector locus (shaded in red). Approximately one third of strains collected from A. thaliana lack the canonical T3SS [23]. (PDF) [file pone.0184195.s004.pdf]
