# Supplementary material for: Similar levels of gene content variation observed for Pseudomonas syringae populations extracted from single and multiple host species
Source: PLoS One. 2017 Sep 7;12(9):e0184195. doi: 10.1371/journal.pone.0184195 (PMC5589212; doi:10.1371/journal.pone.0184195)
Supplement: S1 Table — Information was not available for several strains (listed as "NA"). When non-specific isolation information was provided by collector (such as country of origin), the latitude of country is provided. LMC is an abbreviation for Lake Michigan College. (PDF) [file pone.0184195.s005.pdf]

Table S1: **Location of isolation for strains analyzed in this study.** Information was not available for several strains (listed as "NA"). When non-specific isolation information was provided by collector (such as country of origin), the latitude of country is provided. LMC is an abbreviation for Lake Michigan College.

**Strain**	**Host**	**Location of Isolation**	**Latitude**	**Longitude**	**Reference**
Pph1448a	French bean	Ethiopia	9.50	36.90	(Joardar *et al.*, 2005)
PsyB728a	Snap bean	Wisconsin, USA	44.5	-89.50	(Feil *et al.*, 2005)
PtoDC3000	Tomato	Channel Islands, UK	49.47	2.58	(Buell *et al.*, 2003)
PtoMax13	Tomato	France	46.00	2.00	Unpubl.
PtoNCPPB1108	Tomato	UK	53.11	2.58	(Cai *et al.*, 2011)
Pmp	Plum	NA	NA	NA	(Baltrus *et al.*, 2011)
Pla107	Cucumber	Gunma, Japan	36.37	139.12	(Baltrus *et al.*, 2011)
Pmo	Mulberry	NA	NA	NA	(Baltrus *et al.*, 2011)
Pja	Barley	Tochigi, Japan	36.52	139.82	(Baltrus *et al.*, 2011)
PpiR6	Pea	France	46.00	2.00	(Baltrus *et al.*, 2011)
Pma	Radish	NA	NA	NA	(Baltrus *et al.*, 2011)
Pan	Kiwi	NA	NA	NA	(Baltrus *et al.*, 2011)
Pla106	Cucumber	Shizuoka, Japan	34.97	138.37	(Baltrus *et al.*, 2011)
Ptt	Sugar beet	NA	NA	NA	(Baltrus *et al.*, 2011)
Pac	Maple tree	USA	40.42	-98.7372	(Baltrus *et al.*, 2011)
Pta	Tobbacco	NA	NA	NA	(Baltrus *et al.*, 2011)
PgyB076	Soy bean	Champaign, IL, USA	40.12	-88.24	(Qi *et al.*, 2011)
PgyR4	Soy bean	NA	NA	NA	(Baltrus *et al.*, 2011)
Pae	Horse chestnut	India	21.77	78.87	(Baltrus *et al.*, 2011)
T1	Tomato	Canada	56.76	-86.42	(Almeida *et al.*, 2008)
K40	Tomato	Virginia, USA	37.67	-78.61	(Cai *et al.*, 2011)
Por1-6	Rice	NA	NA	NA	(Reinhardt *et al.*, 2009)
DM2.1.12.02A	Mouse-ear cress	Jasper County, IN, USA	41.19	-87.11	This study
LMC.P10	Mouse-ear cress	LMC, MI, USA	42.10	-86.39	This study
LMC.P80	Mouse-ear cress	LMC, MI, USA	42.10	-86.39	This study
LMC.P91	Mouse-ear cress	LMC, MI, USA	42.10	-86.39	This study
KN2.a.3	Mouse-ear cress	Starke County, IN, USA	41.27	-86.63	This study
Knox623a	Mouse-ear cress	Starke County, IN, USA	41.27	-86.63	This study
Knox652c	Mouse-ear cress	Starke County, IN, USA	41.27	-86.63	This study
Nl.P123	Mouse-ear cress	North Liberty, USA	41.53	-86.43	This study
NP29.1a	Mouse-ear cress	Berrien County, MI, USA	42.09	-86.33	This study
LP217a	Mouse-ear cress	La Porte, Indiana, USA	41.69	-86.85	This study
LP221b	Mouse-ear cress	La Porte, Indiana, USA	41.69	-86.85	This study
LP868.1a	Mouse-ear cress	La Porte, Indiana, USA	41.69	-86.85	This study
RM.P66	Mouse-ear cress	Route Marker, USA	41.36	-86.74	This study
RMX.24.a.1	Mouse-ear cress	Berrien County, MI, USA	42.04	-86.51	This study
RM.P20	Mouse-ear cress	Route Marker, USA	41.36	-86.74	This study
RMX815.1a	Mouse-ear cress	Berrien County, MI, USA	42.04	-86.51	This study
ME812.2b	Mouse-ear cress	Michigan Extension site, USA	42.09	-86.36	This study
LP205a	Mouse-ear cress	La Porte, Indiana, USA	41.69	-86.85	This study
