# Supplementary material for: Similar levels of gene content variation observed for Pseudomonas syringae populations extracted from single and multiple host species
Source: PLoS One. 2017 Sep 7;12(9):e0184195. doi: 10.1371/journal.pone.0184195 (PMC5589212; doi:10.1371/journal.pone.0184195)
Supplement: S2 Table — This table shows the position in the B728a genome of each of the genes unique to group II. Fifty-six genes were conserved in 90% or more of the strains in group II, but absent outside group II. 29% of these genes lie in the syringomycin/syringopeptin biosynthetic cluster, highlighted in Cyan. (PDF) [file pone.0184195.s006.pdf]

Table S2: Genes unique to and conserved in group II. This table shows the position in the B728a genome of each of the genes unique to group II. Fifty six genes were conserved in 90% or more of the strains in group II, but absent outside group II. 29% of these genes lie in the syringomycin/syringopeptin biosynthetic cluster, highlighted in Cyan.

Start	End	Function
419054	419746	Polyhydroxyalkanoate granule-associated protein PhaF
871698	870682	Gluconolactonase (EC 3.1.1.17)
872972	873892	transcriptional regulator, LysR family
874861	874418	hypothetical protein
875334	874876	FIG00963335: hypothetical protein
996403	995840	acetyltransferase, GNAT family
1023614	1023483	FIG00959449: hypothetical protein
1030545	1029403	L-lactate dehydrogenase (EC 1.1.2.3)
1030676	1031572	LysR family regulatory protein CidR
1281770	1281967	FIG00958976: hypothetical protein
1566775	1566948	FIG00966404: hypothetical protein
1734939	1735520	DedA family inner membrane protein YohD
1755166	1754198	luciferase family protein
1892673	1893737	FOG: GGDEF domain
2547726	2546254	Outer membrane component of tripartite multidrug resistance system
2547814	2548428	Transcriptional regulator, TetR family
2548506	2549609	RND efflux membrane fusion protein
2688569	2688441	hypothetical protein
2730832	2731044	FIG00957523: hypothetical protein
2805336	2805578	lipoprotein, putative
3056307	3055600	regulatory protein, LuxR
3057159	3058328	Macrolide-specific efflux protein MacA
3058574	3059179	FIG00957335: hypothetical protein
3060160	3060008	FIG00956999: hypothetical protein
3061643	3060225	RND efflux system, outer membrane lipoprotein CmeC
3091068	3062695	Long-chain-fatty-acid–CoA ligase (EC 6.2.1.3)
3092679	3091468	Predicted hydrolases or acyltransferases (alpha/beta hydrolase superfamily)
3093683	3092751	CmaB
3095545	3093701	Long-chain-fatty-acid–CoA ligase (EC 6.2.1.3)
3095877	3096938	Non-ribosomal peptide synthetase modules, pyoverdine
3097019	3098719	ABC-type hydroxamate-type ferric siderophore transporter, ATP-binding protein
3172404	3173561	Macrolide-specific efflux protein MacA
3173564	3175525	Macrolide export ATP-binding/permease protein MacB (EC 3.6.3.-)
3177074	3175740	Siderophore biosynthesis diaminobutyrate–2-oxoglutarate aminotransferase (EC 2.6.1.76)

Continued on next page

Table S2 – Continued from previous page

Start	End	Function
3177351	3178781	Outer membrane component of tripartite multidrug resistance system
3178778	3179881	PUTATIVE CATION-EFFLUX SYSTEM SIGNAL PEPTIDE PROTEIN
3206174	3205149	Homoserine O-acetyltransferase (EC 2.3.1.31)
3243182	3242289	LysR family transcriptional regulator clustered with dicarboxylate transport
3243314	3244606	Uncharacterized transporter, similarity to citrate transporter
3244607	3245971	Protein of unknown function DUF1446
3245968	3246330	Small uncharacterized protein Bpro4170
3263539	3262145	Uncharacterized iron-regulated membrane protein; Iron-uptake factor PiuB
3263836	3263648	hypothetical protein
3432085	3431795	FIG00960182: hypothetical protein
3763830	3763711	hypothetical protein
3813654	3813932	Cold shock protein CspD
4298907	4298794	hypothetical protein
4316808	4316446	competence protein, putative
4501194	4501826	thioredoxin domain protein
4785405	4785205	FIG00955586: hypothetical protein
4938236	4938102	FIG00960679: hypothetical protein
5030649	5030455	conserved domain protein
5231095	5231568	Conserved domain protein
5258977	5259249	FIG00955051: hypothetical protein
5276341	5275643	FIG00959965: hypothetical protein
5600755	5594942	hypothetical protein
